# Integral Assessment of Species of the Genus *Allium* L. (Amaryllidaceae) in the Western Part of the Kyrgyz Alatau

**DOI:** 10.3390/plants14182890

**Published:** 2025-09-18

**Authors:** Polina V. Vesselova, Gulmira M. Kudabayeva, Daulet Sh. Abdildanov, Bektemir B. Osmonali, Serikbay Ussen, Mikhail V. Skaptsov, Nikolai Friesen

**Affiliations:** 1 Institute of Botany and Phytointroduction, 36D Timiryazev Str., Almaty 050040, Kazakhstan; pol_ves@mail.ru (P.V.V.); kgm_anita@mail.ru (G.M.K.); be96ka_kz@mail.ru (B.B.O.); ussen.s@mail.ru (S.U.); 2 Faculty of Biology and Biotechnology, Al-Farabi Kazakh National University, al-Farabi Ave 71., Almaty 050040, Kazakhstan; 3 South Siberian Botanical Garden, Altai State University, Lesosechnaya Str. 25, 656906 Barnaul, Russia; mr.skaptsov@mail.ru; 4 Botanical Garden, Osnabruck University, Albrechtstrasse 29, 49076 Osnabruck, Germany; nikolai.friesen@uni-osnabrueck.de

**Keywords:** genus *Allium*, ITS data, flow cytometry, Kyrgyz Alatau

## Abstract

This paper presents the results of the current present species composition of the genus *Allium* L. (Amaryllidaceae) of the western part of the Kyrgyz Alatau. The phylogeny, ploidy, and chorological data are presented, and a checklist of species of the genus *Allium* in the study area is compiled. The large subgenera of *Allium*, including *Melanocrommyum*, have been identified, reflecting their prevalence in the mountainous regions of Central Asia. Ploidy of some species (*A. artrosanguineum* and *A. turkestanicum*) of the genus Allium growing in the western part of the Kyrgyz Alatau were studied for the first time using flow cytometry methods. The nrITS sequencing was used to construct a phylogenetic tree including sequences from the NCBI database. The phylogenetic tree of species of the genus Allium of the Kyrgyz Alatau was compiled taking into account previously published data. The list of studied *Allium* species in the Kyrgyz Alatau is represented by 25 species, which include seven subgenera and 17 sections. Also, the nomenclature of onion species is brought to the modern understanding of some species names. Based on specimens of the genus *Allium* collected in the western part of the Kyrgyz Alatau, an integral assessment of the current species composition using molecular–genetic, cytometric, and traditional botanical methods was carried out.

## 1. Introduction

The genus *Allium* L. (Amaryllidaceae J. St.-Hil.), which is a complex systematic group, includes more than 1000 species [1,2]. The ecological range of *Allium* representatives extends from the alpine and subalpine belts to deserts. Almost one third of them grow in the mountains of Central Asia—the world’s largest centre of diversity of *Allium* species. Only in the Tien Shan mountains, which are characterised by a high degree of endemism, there are 13 major subgenera of the genus *Allium*, including 56 species endemic to this mountain system [3,4]. According to the literature data, there are from 9 to 21 endemic species of this genus and 14 subendemic taxa in the flora of Kazakhstan [5,6,7]. The Kyrgyz Alatau is located at the junction of the Northern and Western Tien Shan and characterised by a correspondingly transitional composition of the flora.

**Figure 1:**
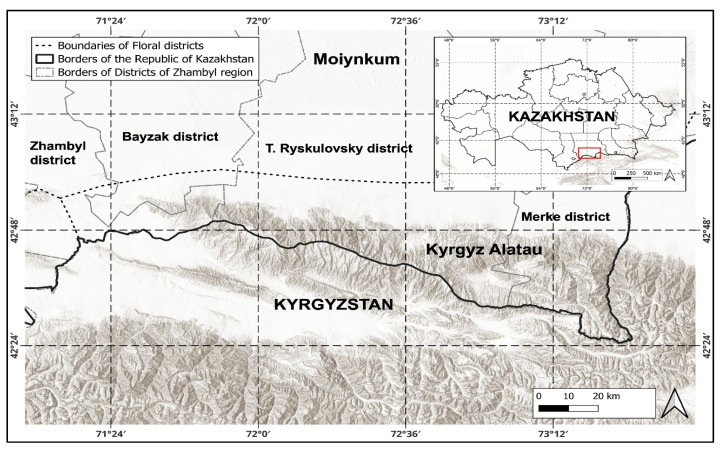
The study area is the Kazakh part of the Kyrgyz Alatau range.

According to the botanical and geographical zoning of Kazakhstan, the Kyrgyz Alatau belongs to the Saharan–Gobi desert region, Iran-Turan subregion, Dzungar-North Tien Shan province, and Kyrgyz mountain subprovince [8] (Figure 1).

In one of the fundamental works on the genus *Allium*, the *Flora of the USSR* [5], the genus was divided into nine sections. In the *Flora of Kazakhstan* [6], for the territory of the republic as a whole, 108 species of Alliums are indicated, 29 of which are stated as endemic. According to the data of *Flora of Kazakhstan Vol. 2. Generic composition of the flora* [9], the genus *Allium* in the flora of Kazakhstan is represented by 140 species.

The list of representatives of the *Allium* genus in the flora of Kazakhstan, published in *Plantarium* [10], includes 128 species. At that, 25 species from seven subgenera and 17 sections are represented in the Kazakh part of the Kyrgyz Alatau range. The Kyrgyz Alatau is a transboundary range; it is possible to expand the list by onion species growing in the territories bordering Kyrgyzstan.

The aim of this study was to conduct a phylogenetic and taxonomic assessment of species of the genus *Allium* L. found in the Kyrgyz Alatau, using data on the internal transcribed spacer (ITS) sequence in order to clarify the systematic position of individual taxa within modern classification systems.

## 2. Results

### 2.1. Distribution Analyses

As a result of the analysis of the literature data, herbarium materials, and our own field research, an annotated checklist of 25 species of the genus *Allium* growing in the Kyrgyz Alatau was compiled. Among the identified species, one is endemic (*A. oreoprasoides*); two subendemic (*A. margaritae*, *A. trachyscordum*), which are also distributed in Kyrgyzstan; and one is a rare species listed in the Red Book of the Republic (*A. suworowii*) [11,12].

**Figure 2:**
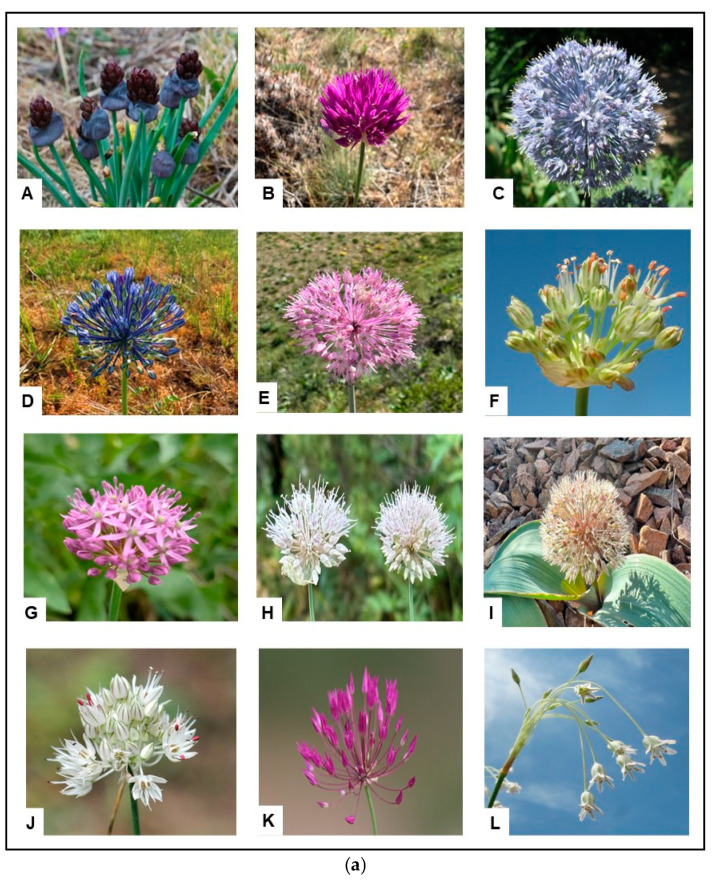

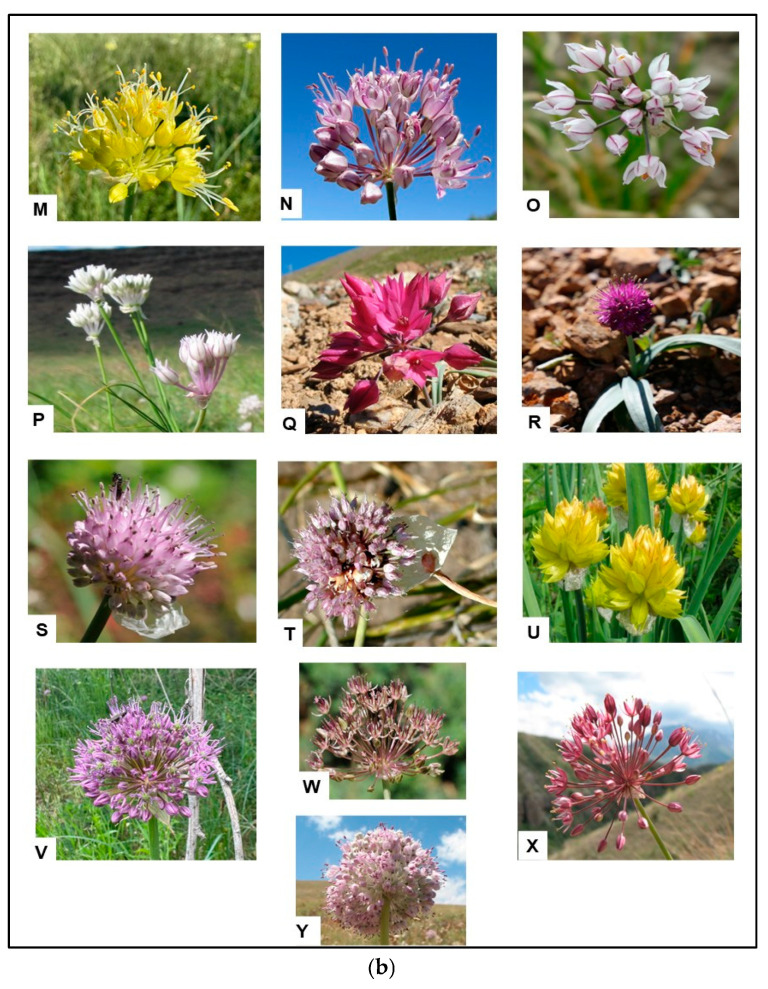
(**a**) Photographs of *Allium* species from the Kyrgyz Alatau. A—*A. atrosanguineum* (photo N. Friesen), B—*A. barsczewskii* (photo Abdildanov), C—*A. caeruleum* [18], D—*A. caesium* (photo Abdildanov), E—*A. caricifolium* (photo Abdildanov), F—*A. dasyphyllum* [19], G—*A. fetisowii* (photo N. Friesen), H—*A. hymenorhizum* (photo Abdildanov), I—*A. karataviense* (photo Abdildanov), J—*A. leptomorphum* [20], K—*A. longiradiatum* [21], L—*A. margaritae* [22]. (**b**) M—*A. obliquum* (photo Abdildanov), N—*A. oreoprasoides* [23], O—*A. oreoprasum* (photo N. Friesen), P—*A. parvulum* [24], Q—*A. platyspathum* (photo Vesselova), R—*A. platystemon* [25], S—*A. polyphyllum* [26], T—*A. sativum* (photo N. Friesen), U—*A. semenowii* [27], V—*A. suworowii* (photo Abdildanov), W—*A. taschkenticum* [28], X—*A. trachyscordum* [29], Y—*A. turkestanicum* [30].

Three species of Alliums, which, according to the literature data [13], were cited for the Kazakhstan part of the Kyrgyz Alatau (*Allium altissimum*, *Allium schoenoprasum*, *A. kokanicum*), were excluded from the list, because no herbarium specimens for these three species from the Kyrgyz Alatau exist, as well as no references in the literature and no image evidence in Internet sources [14,15]. One of the largest subgenera of the studied genus includes two main groups: subgenus *Allium*, consisting of six sections, and subgenera *Melanocrommyum* and *Reticulatobulbosa*, including three sections. All species of the genus *Allium* growing in the western part of the Kyrgyz Alatau are presented in the illustrations provided in Figure 2a,b. For two species, new names have been given: *Allium pallasii* s. l. is divided into two species—*Allium caricifolium* is distributed west of Lake Saisan and in the southeast and south Kazakhstan and Kyrgyzstan, and *A. pallasii* is distributed in northern Kazakhstan to the Siberian Altai [16]. *Allium carolinianum* is also divided into two species—*Allium polyphylum* is now valid for Tianshan and Dzhungar Alatau; *A. carolinianum* is only distributed in the Himalayas [17].

Meanwhile, in other subgenera, several large sections are also distinguished, each with an equal number of species: *Acmopetala* (Subgenus *Melanocrommyum*) and *Falcatifolia* (Subgenus *Polyprason*).

An annotated checklist of the species of the genus *Allium* currently found in the Kyrgyz Alatau is presented below.

**Subgenus *Allium.***
**Section *Allium.***
  ***Allium sativum* L.** (1753 in Sp. Pl. 1: 296)—syn. *A. longicuspis* Regel (1875 in Trudy Imp. S.-Peterburgsk. Bot. Sada 3.2: 45).
  The wild plants described as *A. longicuspis* Regel are feral cultivated garlic [31].
  Perennial. Grows in shrubs and along the banks of streams at the bottom of gorges in the lower belt of mountains. Flowering VI–VIII.
  Examined herbarium specimens from the Kyrgyz Alatau: AA (Geographic area: the southwestern extremity of the Kyrgyz range, the foothills to the northwest, from Almaly-sai, along the meadow slopes, date 03.VI.1963, author Goloskokov V. P.).
  Distribution in Kazakhstan: 25. Zailiyskiy-Kungei Alatau, 25a. Ketmen-Terskey Alatau, 26. Chu-Ili mountains, 27. Kyrgyz Alatau, 28. Karatau, 29. Western Tien-Shan.
  General distribution: Iranian–mountain–Middle Asian cultivated species (cultivated worldwide).

**Section *Brevispatha* Valsecchi** (1974 in Giorn. Bot. Ital. 108(1–2): 92).
  Type species *Allium parciflorum* Viv.
  ***Allium margaritae* B. Fedtsch**. (1918 in Izv. Glavn. Bot. Sada R.S.F.S.R. 18: 14.).
  Perennial. Grows on rubbly outcrops of variegated rocks. Flowering V–VI.
  Examined herbarium specimens from the Kyrgyz Alatau: **AA** (Geographic area: Kyrgyz Alatau, Zhambyl region, Ulken Almalysay gorge, southern slope. Coordinates: N 42°53′56″ E 71°42′43″ altitude—h-1200 m, date: 26.VIII.2024, authors: Vesselova P.V., Bilibayeva B.K., Abdildanov D.Sh., Ussen S., Akhmetzhanova R.K.).
  Distribution in Kazakhstan: 16. Betpakdala, 26. Chu-Ili mountains, 27. Kyrgyz Alatau.
  General distribution: Betpakdala-North Tyanshan (endemic).

**Section *Caerulea* (Omelcz.) F. O. Khass.** (1996 in Öztürk, Seqmen & Gork (eds.) Plant Life in South-West and Central Asia: 150).
  Type species *Allium caeruleum* Pall.
  ***Allium caesium* Schrenk** (1844 in Bull. Cl. Phys.-Math. Acad. Imp. Sci. Saint-Pétersbourg 2: 113).
  Perennial. Grows in steppes and deserts, on plumes and mountain slopes up to the middle belt. Flowering IV–VII.
  Examined herbarium specimens from the Kyrgyz Alatau: **MW** (foothills of the Kyrgyz range, feather grass steppe, 08. VI. 1958, collector Gubanov I.; northwestern extremity of the Kirghiz range, Syugaty gorge in the lower tichi of the river, along the southern rocky slopes, 05. VI. 1963, collector: Goloskokov V.P.); **AA** (Western tip of the Kyrgyz Alatau, valley of the Merke River, left-bank slope among bushes. 29.V.1985, Chubarova T.U. Northwestern Tip of the Kirghiz Range. Syugata Gorge in the lower reaches of the river. Along the southern rocky slopes.05.VI.1963. Goloskokov V.P. Kyrgyz Alatau, Kyzylsu river gorge, foothills, northeastern slopes, altitude –1500 m 16.VII.1948. Rubtsov N. I. Kyrgyz range. Almalyk-sai gorge, a gap west of the beginning of the gorge, feather grass steppe with spirea bushes and groups of St. John’s wort. 25.V.1961. Fisyun V.V. Chibindy Gorge, range crest, near the rocks. 04.VI.1960. Gamayunova A.P. Zhambyl region, Turar, Ryskulovsky district, Makpal gorge N 42.8651568 E 71.9170940 03.V.2025. Vesselova P.V., Kudabayeva G.M., Friesen N.V., Abdildanov D.Sh., Kenesbay A.Kh., Akhmetzhanova R.K.).
  Distribution in Kazakhstan: 3. Irtysh, 9. Turgai, 10. Western Shallow Soil, 11. Eastern Cretaceous-Popochon, 16. Betpakdala, 17. Moyinkum, 18. Balkhash-Alakol, 21. Turkestan, 24. Dzungarian Alatau, 25. Zailiyskiy-Kungei Alatau, 25a. Ketmen-Terskey Alatau, 26. Chu-Ili Mountains, 27. Kyrgyz Alatau, 28. Karatau, 29. Western Tien Shan.
  General distribution: North-Turanian–Mountain–Middle Asian (Kazakhstan, Kyrgyzstan, Tajikistan, Uzbekistan, Xinjiang).

***Allium caeruleum* Pall.** (1773 Reise Russ. Reich 2: 737).
  Perennial. Grows on high dry steppes, in foothills and mountains, in wet meadows and shrubs. Flowering V–VI.
  Examined herbarium specimens from the Kyrgyz Alatau: MW (Akyrtobe, Taldybulak gorge, date 11.VII. 1924, authors: Popov M.G., Makieva E.M.); AA (Kara-Bulak gorge, the bottom of the gorge, among the high mint. date 07.VI.1961 author: Gamayunova A. P. Geographic area: Syugates. A damp meadow along the bottom of the gorge in the upper reaches. date 03.VI.1961, author: Gamayunova A.P. Geographic area: Southwestern Extremity of the Kyrgyz ange. Almaly-Sai gorge. Along the southern rocky slopes. date 03.VI.1963. Goloskokov V. P. Geographic area: Right side of the sh.r. Merke. Southwestern rocky slope. date 03.VII.1947, authors: Rubtsov N.I., Stepanova E.F.).
  Distribution in Kazakhstan: 2. Tobyl-Ishim, 4. Semipalatinsk hog, 9. Turgai, 10. Western Shallow Soil, 11. Eastern Shallow Soil, 12. Zaisan, 16. Betpakdala, 18. Balkhash-Alakol, 24. Dzungarian Alatau, 25. Zailiyskiy-Kungei Alatau, 25a. Ketmen-Terskey Alatau, 26. Chu-Ili Mountains, 27. Kyrgyz Alatau.
  General distribution: Central Palaearctic (Russia: Altai, Kazakhstan, Kyrgyzstan, Tajikistan, Uzbekistan, China: Xinjiang).

**Section *Pallasia* (Tzag.) F.O.Khass., R.M. Fritsch & N. Friesen (2017 in Flora Uzbekistana 1: 87).** Type species *A. pallasii* Murrai.
  ***Allium caricifolium* Kar et Kir.** (1841 in Bull. Soc. Imp. Naturalistes Moscou 14: 854)—*Allium pallasii* Murrai pro parte [16].
  Perennial. Grows on rubbly and stony slopes, outcrops of variegated rocks, less often in the upper belts of mountains. Flowering V–VI.
  Examined herbarium specimens from the Kyrgyz Alatau: AA (Zhambyl region, Merken district, Molaly gorge. Coordinates: N 42°45′35″ E 73°1′29″ altitude h-1230 m. date 24.V.2023. authors: Vesselova P.V., Kudabayeva G.M., Shormanova A.A., Osmonali B.B., Abdildanov D.Sh., Ussen S.; Geographic area: Zhambyl region, Turar Ryskulov district, Makpal gorge. Coordinates: N 42.8651568 E 71.9170940, date 03.V.2025. authors: Vesselova P.V., Kudabayeva G.M., Friesen N.V., Abdildanov D.Sh., Kenesbay A.Kh., Akhmetzhanova R.K.).
  Distribution in Kazakhstan: 24. Dzungarian Alatau, 25. Zailiyskiy-Kungei Alatau, 25a. Ketmen-Terskey Alatau, 26. Chu-Ili Mountains, 27. Kyrgyz Alatau.
  General distribution: mountain–central Asian (Kazakhstan, Kyrgyzstan, Tajikistan, Uzbekistan, China: Xinjiang).

**Section *Mediasia* F. O. Khass., Yengal. & N. Friesen (2006 in Aliso 22: 386).**
  Type species *A. turkestanicum* Regel.
  ***Allium turkestanicum* Regel** (1875 in Trudy Imp. S.-Peterburgsk. Bot. Sada 3.2: 197).
  Perennial. Grows in clay, sandy and rubbly desert steppes. Flowering VI–VII.
  Examined herbarium specimens from the Kyrgyz Alatau: AA (Geographic area: Kyrgyz range, northern gorges, Katudzhan gorge, dry slopes of the left bank; on shallow earth in groups. date 16.VI.1961 author: Gamayunova A.P.).
  Distribution in Kazakhstan: 15. Kyzylorda, 16. Betpakdala, 18. Balkhash-Alakol, 21. Turkestan, 25. Zailiyskiy-Kungei Alatau, 26. Chu-Ili Mountains, 27. Kyrgyz Alatau, 28. Karatau.
  General distribution: North Turanian–mountain–Middle Asian (Kazakhstan, Kyrgyzstan, Tajikistan, Turkmenistan, Uzbekistan).

**Section *Minuta* F. O. Khass.** (1996 In Öztürk, Seqmen & Gork (eds.) Plant Life in South-West and Central Asia: 150).
  Type species *A. minutum* Vved.
  ***Allium parvulum* Vved.** (1934 in Byull. Sredne-Aziatsk. Gosud. Univ. xix. 124).
  Perennial. Grows on rubbly variegated plumes of mountains. Flowering V–VI.
  Examined herbarium specimens from the Kyrgyz Alatau: **AA** (Geographic area: Dzhambi region, steppe hillock between Chu and Lugovaya stations. date 25.V.1948. Pavlov N.V. Geographic area: South-Kazakh region. Department of Land Management, Aulie-Ata district. Plain from Ak-Chulak. Bluegrass-sedge steppe with coarse forbs. date 14.V.1933, author: Kornilova V.S.).
  Distribution in Kazakhstan: 25. Zailiyskiy-Kungai Alatau, 27. Kyrgyz Alatau, 28. Karatau.
  General distribution: Prityanshansky (Kazakhstan, Kyrgyzstan).

**Subgenus Butomissa (Salish.) N. Friesen** (2006 in Aliso 22: 387)—Genus Butomissa Salisb. 1866 in Gen. Pl. fragm. Cont. part. Liriogamae: 90.
  **Section *Austromontana* N. Friesen** (2006 in Aliso 22: 387).
  Type species *Allium oreoprasum* Schrenk.
  ***Allium oreoprasum* Schrenk** (1842 in Bull. Acad. Imp. Sci. Saint-Pétersbourg 10.23: 354).
  Perennial. Inhabits rocks and rocky slopes of mountains. Flowering V–VII.
  Examined herbarium specimens from the Kyrgyz Alatau: **AA** (Geographic area: Kyrgyz Alatau, valley of the Merke River, right side of the Taldysu tract, southern slope. date 26.VI.1985. author: Chubarova T.U. Geographic area: Western Extremity of the Kyrgyz Alatau, Kaindy River Valley, Dry Eastern Rocky Slope in the Lower Cordon Area. date 20.V.1984. author: Nelina N.V. Geographic area: West part of the Kyrgyz Alatau, southern. macroslope. the valley of the Nelda River. Rocky slope of the southeastern exposure, thickets. date 21.VI.1984. author: Nelina N.V.).
  Distribution in Kazakhstan: 24. Dzungarian Alatau, 25. Zailiyskiy-Kungei Alatau, 25a. Ketmen-Terskey Alatau, 27. Kyrgyz Alatau, 29. Western Tien Shan.
  General distribution: Mountain–central Asian (Afghanistan, Kazakhstan, Kyrgyzstan, Pakistan, Tajikistan, Tibet, Uzbekistan, Western Himalayas, Xinjiang).

**Subgenus *Cepa* (Mill.) Prokh. (1990** in Razp. Slov. Akad. Znanosti Umetn., Razr. Nar. Vede (SAZU) 31: 250, 251)—*Cepa* Mill. 1754 in Gard. Dict. Abr., ed. 4.
  Type species *Allium cepa* L.
  **Section *Annuloprason* T.V. Egorova** (1977 in Rast. Tsent. Azii, Mater. Bot. Inst. Komarova 7: 57).
  Type species *Allium atrosanguineum* Schrenk.
  ***Allium atrosanguineum* Schrenk** (1842 in Bull. Acad. Imp. Sci. Saint-Pétersbourg 10.23: 355).
  Perennial. Grows on stony and fine-grained places in alpine and subalpine belts of mountains, often in huge thickets. Flowering VI–VIII.
  Examined herbarium specimens from the Kyrgyz Alatau: **AA** (Geographic area: Western part of the Kyrgyz Alatau, Southern macroslope, Kenkol valley, date 04.VI.1984. author: Karmysheva N.Kh. Geographic area: Western extremity of the Kyrgyz Alatau, the valley of the Aspara river, the Kumbel Pass, a wet meadow and a southeastern stony–rubble slope. date 12.VII.1984. author: Zaripov R.G. Geographic area: Kyrgyz range, upper reaches of the gorge. Solver. Alpine lawn on compacted soil. date 17.VI.1961. Fisyun V.V. Geographic area: Kyrgyz Alatau range, Zhambyl region, Turar Ryskulov district, Karakystak. Coordinates: N 42°40′7″ E 72°50′26″ altitude—h-2530m. date 19.07.2023. authors: Vesselova P.V., Kudabayeva G.M., Friesen N.V., Osmonali B.B., Abdildanov D.Sh., Ussen S.).
  Distribution in Kazakhstan: 23. Tarbagatai, 24. Dzungarian Alatau, 25. Zailiyskiy-Kungei Alatau, 25a. Ketmen-Terskey Alatau, 27. Kyrgyz Alatau, 29. Western Tien Shan.
  General distribution: mountain–central Asian (Afghanistan, China, Kazakhstan, Kyrgyzstan, Pakistan, Tajikistan, Uzbekistan).
  ***Allium semenovii* Regel** (1986 in Bull. Soc. Imp. Naturalistes Moscou 41.1: 449).
  Perennial. Grows in alpine meadows. Flowering VI–VII.
  Examined herbarium specimens from the Kyrgyz Alatau: **AA** (Geographic area: Kyrgyz range, Chungur Gorge, upper reaches of the Solyusher river, date 17.VI.1961. author: Fisyun V.V.).
  Distribution in Kazakhstan: 24. Dzungarian Alatau, 25. Zailiyskiy-Kungei Alatau, 25a. Ketmen-Terskey Alatau, 27. Kyrgyz Alatau.
  General distribution: Mountain–Middle Asian–Himalayan (Kazakhstan, Kyrgyzstan, Tajikistan, Western China, India: Himalayas).

**Subgenus *Melanocrommyum* (Webb & Berth.) Rouy** (1910 in Fl. France 12: 378)—*Allium* sect. *Melanocrommyum* Webb et Berth (1846 in Phytogr. Canar. 3: 347).
  Type species *Allium nigrum* L.
  **Section *Acmopetala* R.M. Fritsch** (1992 in Hanelt et al. (eds.), The genus *Allium*: taxonomical problems and genetic resources: 74).
  Type species *Allium backhousianum* Regel.
  ***Allium dasyphyllum* Vved.** (1925 in Byull. Sredne-Aziatsk. Gosud. Univ. ix. Suppl. 6).
  Perennial. Grows on stony slopes in the upper belt of mountains. Flowering VII.
  Distribution in Kazakhstan: 27. Kyrgyz Alatau [32].
  General distribution: Kyrgyz-Alatau (endemic to the Kyrgyz Alatau (Kyrgyzstan, Kazakhstan—subendemic).
  ***Allium taschkenticum* F.O. Khass. et R.M. Fritsch** (1994 in Linz. Biol. Beitr. 26: 971).
  Perennial. Grows on shrubby and herbaceous slopes in the lower belt of mountains. Flowering V–VI.
  Distribution in Kazakhstan: 26. Chu-Ili Mountains, 27. Kyrgyz Alatau, 29. Western Tien Shan.
  *A. taschkenticum* occurs in the Kyrgyz Alatau [33], but so far, we have not seen any herbarium specimens.
  General distribution: Western Tien Shan (Kazakhstan—subendem, Kyrgyzstan, Uzbekistan).
  ***Allium suworowii* Regel** (1881 in Gartenflora 30: 356).
  Perennial. Grows on shrubby and herbaceous slopes and plumes of mountains. Flowering V–VI.
  Examined herbarium specimens from the Kyrgyz Alatau: **AA** (Geographic area: A saline damp meadow in the steppe between Chu and Lugovaya stations. date 24.V.1948. author: Pavlov N.V. Geographic area: Kyrgyz Alatau, the valley of the Kaindy river, near the road in the vicinity of the lower cordon. date 20.V.1984. author: Nelina N.V. Geographic area: Kyrgyz Alatau, uroch. Chungur (Slutorsky) gorge Kara-Archa. date 28.V.1984. author: Nelina N. V. Geographic area: Zhambyl Region, Turar Ryskulov district, Makpal gorge. Coordinates N 42.8651568 E 71. 9170940 altitude h-1182 m. date 03.V.2025. authors: Vesselova P.V., Kudabayeva G.M., Friesen N.V., Abdildanov D.Sh., Kenesbay A.Kh., Akhmetzhanova R.K.).
  Distribution in Kazakhstan: 18. Balkhash-Alakol, 21. Turkestan, 27. Kyrgyz Alatau, 28. Karatau, 29. Western Tien Shan.
  General distribution: Near-North Tien Shan (Afghanistan, Kazakhstan, Kyrgyzstan, Tadzhikistan, Turkmenistan, Uzbekistan).

**Section *Longibidentata* (R.M. Fritsch) R.M. Fritsch** (2009 in Bot. Jahrb. Syst. 127.4: 465)—1994 in in Khassanov & Fritsch, Linzer Biol. Beiträge 26: 974.
  Type species *Allium fetisowi* Regel.
  ***Allium fetisowi* Regel** (1877 in Trudy Imp. S.-Peterburgsk. Bot. Sada 5: 631).
  Perennial. Grows on shrubby and herbaceous slopes in the lower belt of mountains. Flowering V–VI.
  Examined herbarium specimens from the Kyrgyz Alatau: **MW** (Geographic area: Akyrtobe, Taldybulak gorge, subalpine and alpine cereals, date 12. VII. 1924, author: Popov M.G.); **AA** (Geographic area: Western part of the Kyrgyz Alatau, Chungur gorge, right bank of the river, southeastern slope and top of the mountain. 28.VI.1985. Chubarova T.U. Geographic area: Northwestern part of the Kyrgyz range. Syugata gorge in the upper reaches of the river. Along the steep northern slope. date 07.VI.1963. author: Goloskokov V.P. Geographic area: Kyrgyz range, northern gorges. Almalyk-sai gorge, steppe slopes with shrubs, at the top of the spurs, altitude—1500 m, date 23.V.1963. author: Gamayunova A.P. Geographic area: Kyrgyz range, Almalyk-sai gorge. Steppe meadows in the middle part of the range along the wide saddle of the Kaindy. date 01.VI.1961 author: Gamayunova A.P. Geographic area: Kyrgyz range, Almalyk-sai gorge 3rd gap to the west of the main gorge. Steppe slopes of the southern exposure, stony places. date 26.V.1961. author: Fisyun V. V.).
  Distribution in Kazakhstan: 25. Zailiyskiy-Kungei Alatau, 26. Chu-Ili Mountains, 27. Kyrgyz Alatau, 29. Western Tien Shan.
  General distribution: Tien Shan (Kazakhstan, Kyrgyzstan, China).

**Section *Miniprason* R.M. Fritsch** (1992 in Hanelt et al. (eds.), The genus *Allium*: taxonomical problems and genetic resources: 74).
  Type species *Allium karataviense* Regel.
  ***Allium karataviense* Regel** (1875 in Acta Horti Petropolit. 3.2: 243).
  Perennial. Grows on rubbly mobile placers in the lower and middle belts of mountains.
  Flowering IV–VI.
  Examined herbarium specimens from the Kyrgyz Alatau: **AA** (Geographic area: Kyrgyz Range. 03.VII.1947. Rubtsov N. I., Stepanova E. F. Geographic area: Kyrgyz range. The vicinity of the city of Dzhambul, foothills, the beginning of the Butumainak gorge, scree, date 18.V.1961, author: Fisyun V.V. Geographic area: Western part of the Kyrgyz Alatau, valley of the Syugatti River, rocky canyon, thalweg. date 24.V.1984. author: Nelina N. V.).
  Distribution in Kazakhstan: 26. Chu-Ili Mountains, 27. Kyrgyz Alatau, 28. Karatau, 29. Western Tien-Shan.
  General distribution: Iranian–mountain–Middle Asian (Afghanistan, Kazakhstan, Kyrgyzstan, Tajikistan, Uzbekistan).

**Subgenus *Polyprason* Radic** (1990 in Razp. Slov. Akad. Znan. Umet. 31: 253).
  Type species *Allium moschatum* L.
  **Section *Falcatifolia* N. Friesen** (2006 in Aliso 22: 390).
  Type species *Allium carolinianum* DC.
  ***Allium platyspathum* Schrenk** (1841 in Enum. Pl. Nov.:7).
  Perennial. Grows in subalpine and alpine meadows. Flowering VI–VII.
  Examined herbarium specimens from the Kyrgyz Alatau: **AA** (Geographic area: Kyrgyz Alatau. Kaindy tract, grass–sedge bog with birch and spruce, h–2200 m above sea level. date 18.VII.1948. author: Rubtsov N. I. Geographic area: Aspara Forest, Kumbel pass, southeastern slope, altitude—h–3000–3500, date 12.VIII.1980. author: Zaripov R.G. Geographic area: Kyrgyz Alatau, Karabalty gorge, near the Tyuzashu pass. Along the wet sais, 2750 m. date 16.VII.1970. author: Roldugin I. I.).
  Distribution in Kazakhstan: 22. Altai, 23. Tarbagatai, 24. Dzungarian Alatau, 25. Zailiyskiy-Kungei Alatau, 25a. Ketmen-Terskey Alatau, 27. Kyrgyz Alatau, 29. Western Tien Shan.
  General distribution: mountain–central Asian (Afghanistan, Altai, Kazakhstan, Kyrgyzstan, Mongolia, Mongolia, Pakistan, Tajikistan, Uzbekistan, Western China).
  ***Allium polyhyllum* Kar. et Kir.** (1842 in Bull. Soc. Imp. Naturalistes Moscou 15: 509)—syn. *A. carolinianum* Redoute pro parte [17].
  Perennial. Occurs on rubbly slopes, in alpine and subalpine belts of mountains. Flowering VII–VIII.
  Examined herbarium specimens from the Kyrgyz Alatau: **MW** (Geographic area: upper reaches of the Kishi Kaindy, rocky slope, date 19.VII. 1951, author: Golubev V.); **AA** (Geographic area: Kyrgyz range, 4–5 km west of the Merke river gorge. date 18.VII.1947. author: Rubtsov N.I., Stepanova E.F. Geographic area: Northern macroslope, shch. Nelds. Rocky slopes. date 15.VI.1984. author: Nelina N.V. Geographic area: Kyrgyz range, gorge sulyusher. Alpine lawns near snowfields. date 17.VI.1961. author: Fisyun V.V. Geographic area: Kyrgyz Alatau range, Shaldy in the rocks. 27.VIII.1951. author: Baitenov M.S.).
  Distribution in Kazakhstan: 24. Dzungarian Alatau, 25. Zailiyskiy-Kungei Alatau, 25a. Ketmen-Terskey Alatau, 27. Kyrgyz Alatau, 29. Western Tien Shan.
  General distribution: mountain–central Asian (Afghanistan, Kazakhstan, Kyrgyzstan, Mongolia, Nepal, Pakistan, Tajikistan, Tibet, Uzbekistan, China).
  ***Allium hymenorhizum* Ledeb.** (1830 in Fl. Altaica 2:12).
  Perennial. Inhabits damp saline meadows, in mountains on grassy marshes and meadows.
  Flowering VI–VII.
  Examined herbarium specimens from the Kyrgyz Alatau: **AA** (Geographic area: Western part of the Kyrgyz Alatau, valley of the Aspara river, Archaly tract. date 15.07.1984. author: Zaripov R.G. Geographic area: Western part of the Kyrgyz Alatau, the valley of the Aspara river, the Kumbel Pass, a wet meadow. date 16.08.1984. author: Zaripov R.G. Geographic area: Kyrgyz range. The Aral-Tyube river gorge, a gravelly slope among fescue vegetation. date 05.08.1947. authors: Rubtsov N.I., Stepanova E.F. Geographic area: Kyrgyz Alatau range, Zhambyl region, Turar Ryskulov district, serpentine. Coordinates: N 42°40′26″ E72°50′31″ altitude—h-2290m. date 19.07.2023. authors: Vesselova P.V., Kudabayeva G.M., Friesen N.V., Osmonali B.B., Abdildanov D.Sh., Ussen S.).
  Distribution in Kazakhstan: 18. Balkhash-Alakol, 22. Altai, 23. Tarbagatai, 24. Dzungarian Alatau, 25. Zailiyskiy-Kungei Alatau, 25a. Ketmen-Terskey Alatau, 26. Chu-Ili Mountains, 27. Kyrgyz Alatau, 28. Karatau, 29. Western Tien-Shan.
  General distribution: Iranian–mountain–central Asian (Afghanistan, Russia, Iran, Kazakhstan, Kyrgyzstan, Mongolia, Tajikistan, Turkey, Uzbekistan, China).

**Section *Oreiprason* F. Herm.** (1939 in Feddes Repert. 46: 57).
  Type species *Allium saxatile* M.Bieb.
  ***Allium leptomorphum* Vved.** (1952 in Bot. Mater. Gerb. Inst. Bot. Akad. Nauk Uzbeksk. S.S.R. xiii. 29).
  Perennial. Grows on rocks and placers of mountains. Flowering VII–VIII.
  Distribution in Kazakhstan: 25. Zailiyskiy-Kungei Alatau, 26. Chu-Ili Mountains, 27. Kyrgyz Alatau.
  General distribution: North Tianshanian (Kazakhstan, Kyrgyzstan).
  ***Allium obliquum* L.** (1753 in Sp. Pl. 1: 296).
  Perennial. Grows on rocks and stony slopes in foothills and lower belt of mountains. Flowering VI–VII.
  Examined herbarium specimens from the Kyrgyz Alatau: **AA** (Geographic area: Kyrgyz Alatau, Kultakhr gorge, scree, date 19.08.1948. author: Rubtsov N. I.).
  Distribution in Kazakhstan: 23. Tarbagatai, 24. Dzungarian Alatau, 25. Zailiyskiy Kungei, Alatau, 25a. Ketmen-Terskey Alatau, 26. Chu-Ili Mountains, 27. Kyrgyz Alatau, 28. Karatau.
  General distribution: Central Palaearctic (Romania, Ukraine, Russia (Southern Urals, Siberia), Kazakhstan, Kyrgyzstan, Mongolia, China (western).

**Subgenus *Porphyroprason* (Ekberg) R.M. Fritsch (2006 in Aliso 22: 386**—*Allium* sect. *Porphyroprason* Ekberg 1969 in Bot. Not. 122: 65).
  Type species *Allium oreophilum* C.A:Mey.
  **Section *Porphyroprason* Ekberg** (1969 in Bot. Not. 122: 65).
  Type species *A. oreophilum* C. A. Mey.
  ***Allium platystemon* Kar. & Kir.** (1842 in Bull. Soc. Imp. Naturalistes Moscou 15(2): 514).—Syn. *A. oreophillum* C.A.Mey. pro parte [34].
  Perennial. Grows on rubbly slopes in the upper belt of mountains. Flowering VI–VIII.
  Examined herbarium specimens from the Kyrgyz Alatau: MW (Geographic area: Kishi Kaindy, the top of the mountain range, 19.VII. 1951, collector Golubev V.); AA (Geographic area: Kyrgyz Alatau range, 4–5 km west of the Merke river gorge, northern gravelly slope, altitude—2700 m. date 14.VII.1947. authors: Rubtsov N.I., Stepanova E.F. Geographic area: Kyrgyz Alatau. Upper reaches of the Shamsi River, alpine meadow, on crushed stone, date 31.VII.1948. Rubtsov N.I.).
  Distribution in Kazakhstan: 23. Tarbagatai, 24. Dzungarian Alatau, 25. Zailiyskiy-Kungei Alatau, 25a. Ketmen-Terskey Alatau, 27. Kyrgyz Alatau, 29. Western Tien Shan.
  General distribution: mountain–central Asian (Afghanistan, Kazakhstan, Kyrgyzstan, Pakistan, Tajikistan, Uzbekistan, China).

**Subgenus *Reticulatobulbosa* (Kamelin) N. Friesen** (2005 in Aliso 22: 389).
  Type species *Allium lineare* L.
  **Section *Campanulata* Kamelin** (1980 in Bot. Journ. 65.10: 1461).
  Type species *Allium xiphopetalum* Aitch. & Baker.
  ***Allium barsczewskii* Lipsky** (1900 in Acta Horti Petropolit. 18: 114).
  Perennial. Occurs on fine-grained or rubbly steppes, slopes of foothills and mountains.
  Flowering V–VII.
  Examined herbarium specimens from the Kyrgyz Alatau: AA (Geographic area: Aulie-Ata district. Trails of the Kyrgyz range. To the northeast of the Ak-Chulak base of the Konesovkhoz, date 13.V.1933. author: Kornilova V.S. Geographic area: Kyrgyz Alatau Suyundyk-sai river. The northern slope is about 2500 m above sea level. date 22.VII.1947. authors: Rubtsov N. I., Stepanova E. F. Geographic area: Kyrgyz range. Sundyk-sai river gorge, northern slope, altitude 2500 m. date 28.VII.1947. authors: Rubtsov N.I., Stepanova E.F. Geographic area: Dzhamb. region, steppe trail of the mountain between the village of Chulak-Tau and Kok-Tal. date 30.V.1948. Pavlov N.V. Geographic area: Kyrgyz range, western extremity. Butumainak gorge. Limestone cliffs, southern slope. date 19.V.1961. author: Fisyun V.V. Geographic area: Kyrgyz range, northern gorges. Almalyk-sai gorge, southern steppe slopes. date 22.V.1961. author: Fisyun V.V. Geographic area: Kyrgyz Alatau range the central part of Almalyk-say. Southern steppe slope among *Carex pachystilis*. 22.V.1961. author: Gamayunova A.P. Geographic area: Kyrgyz range, Northern Gorges. Almaly-Sai Gorge. Meadow-Steppe Slopes. date 24.V.1961. author: Fisyun V.V. Geographic area: Kyrgyz range, Almalyk-sai gorge, eastern slit. date 24.V.1961 author: Gamayunova A.P. Geographic area: Kyrgyz range, northern gorges. Uzunbulak gorge, ranges of spurs. date 28.V.1961. author: Fisyun V.V. Geographic area: Kyrgyz range, the upper reaches of the Kaindy river, near the top of Mount Tegres, at an altitude of altitude h–1500 m. date 01.VI.1961. author: Gamayunova A. P. Geographic area: Kyrgyz range, northern gorges. Slopes of the Dzhaksalyk-Sai range. date 01.VI.1961. author: Gamayunova A. P. Geographic area: Foothill loess plain of the Kyrgyz range between Lugovoy and Dzhambul. Among the ephemerae. date 15.V.1963. author: Goloskokov V.P. Geographic area: Foothill loess plain of the Kyrgyz range between Merke and Meadow. Among the ephemeral meadow. date 15.V.1963. author: Goloskokov V.P. Geographic area: Southwestern part of the Kyrgyz range. Almaly-sai gorge. Along the southern rocky slopes. date 02.VI.1963. author: Goloskokov V.P. Geographic area: Western part of the Kyrgyz Alatau, valley of the Syugatti river, stony red range of low mountains. date 24.V.1984 author: Nelina N.V. Geographic area: Kyrg. Alat. river Aspara, tract. Char, watershed. date 22.VI.1985. author: Chubarova T.U. Geographic area: Zhambyl region, Kyrgyz Alatau. Merke Gorge, near the outpost date 12.V.2018 authors: Vesselova P.V., Mukhtubayeva S.K., Danilov M.P. Geographic area: Zhambyl region, Kyrgyz Alatau. Merke gorge, near the outpost, date 12.V.2018, authors: Vesselova P.V., Mukhtubayeva S.K., Danilov M.P.).
  Distribution in Kazakhstan: 21. Turkestan, 25. Zailiyskiy-Kungei Alatau, 26. Chu-Ili Mountains, 27. Kyrgyz Alatau, 28. Karatau, 29. Western Tien-Shan.
  General distribution: Iranian–Mountain–Middle Asian–Western Himalayan (Afghanistan, Iran, Kazakhstan, Kirgizstan, Pakistan, Tadzhikistan, Uzbekistan, West Himalaya).
  ***Allium longiradiatum* (Regel)** Vved. (1923 in Vved. & al., Key Fl. Tashkent Pt. 1—*Allium tataricum* var. *longiradiatum* Regel 1875 in Acta Horti Petropolit. 3.2: 180).
  Examined herbarium specimens from the Kyrgyz Alatau: AA (Kyrgyz Alatau range, foothills. date 25.V.1986. author: Kamenetskaya I.I.).
  Perennial. Inhabits loess plains and foothills. Flowering IV–V.
  Distribution in Kazakhstan: 27. Kyrgyz Alatau, 29. Western Tien Shan.
  General distribution: Western Tien Shan (Kazakhstan, Uzbekistan).

**Section *Scabriscapa (Tscholok.) N. Friesen*** (2006 in Aliso 22: 389).
  Type species *Allium scabriscapum* Boiss.
  ***Allium trachyscordum* Vved.** (1925 in Byull. Sredne-Aziatsk. Gosud. Univ. ix. Suppl. 11).
  Perennial. Occurs on rubbly mountain slopes. Flowering VI–VII.
  Examined herbarium specimens from the Kyrgyz Alatau: AA (Geographic area: Southwestern part of the Kyrgyz range, foothills to the northwest of Almaly-Say. date 03.VI.1963. author: Goloskokov V.P. Geographic area: Western part of the Kyrgyz Alatau, dale Sugats, left-bank lowlands from the cordon 2–3 km up, date 15.VI.1985 author: Karmysheva N.Kh. Geographic area: Western part of the Kyrgyz Alatau, northern macroslope, valley of the Syugata river, red hills adjacent to the mouth of the river, date 05.VIII.1988. author: Karmysheva N.Kh.).
  Distribution in Kazakhstan: 16. Betpakdala, 26. Chu-Ili Mountains, 27. Kyrgyz Alatau, 28. Karatau.
  General distribution: Betpakdala-Severoturan-Tianshanian (Kazakhstan, Kyrgyzstan).

**Section *Nigrimontana* N. Friesen** (2006 in Aliso 22:390).
  Type species *Allium drobovii* Vved.
  ***Allium oreoprasoides* Vved.** (1925 in Trudy Turkestansk. Nauchn. Obshch. 2: 29).
  Perennial. Inhabits rubbly mountain slopes. Flowering V–VI.
  Examined herbarium specimens from the Kyrgyz Alatau: AA (Geographic area: The western part of the Kyrgyz Alatau, the valley of the Aspara River, the Archaly tract, date 19.VIII.1984. author: Zaripov R.G. Geographic area: Western part Kyrgyz Alatau, northern macroslope, mouth of the Syugatta river, red-crushed stone ranges, date 24.V.1984. author: Nelina N.V.).
  Distribution in Kazakhstan: 27. Kyrgyz Alatau, 28. Karatau.
  General distribution: Western Tianshan (Kazakhstan (Endem).

The areal analysis of the studied group of species showed that 16 types of ranges were distributed among seven arealological groups: Central Palearctic (two species), Mountain–Central Asian (seven species), Mountain–Middle Asian (five species), North-Turano–Mountain–Middle Asian (two species), Betpakdalin-Tianshan (two species), Tianshan (six species), Prityanshan (two species). Such a distribution of species suggests that most of the species studied originated in the mountains.

### 2.2. Flow Cytometry

For six species collected during expedition trips (*Allium turkestanicum*, *A. suworowii*, *A. barzsczewskii*, *A. artrosanguineum*, *A. margaritae*, *A. caeruleum*), flow cytometry analysis was performed. In addition, literary information on the karyology of species of the genus *Allium* growing in the Kyrgyz Alatau is presented (Table 1).

**Figure 3:**
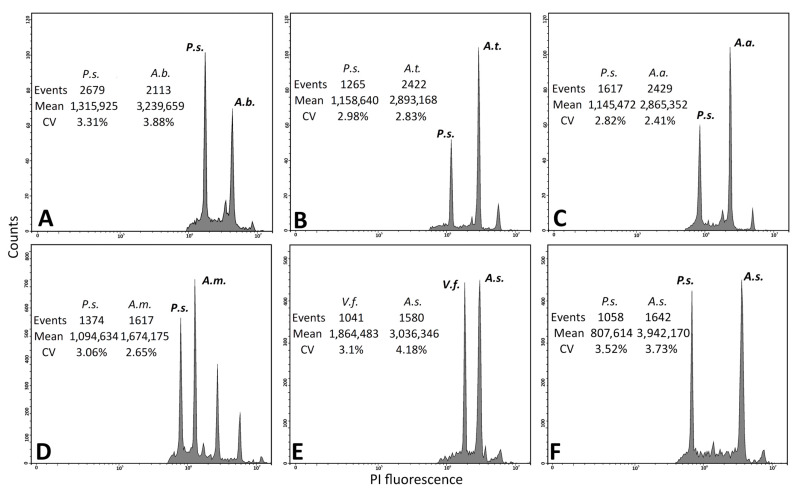
Examples of ungated flow cytometric histograms of the *Allium* samples (log scale). (**A**) *A. barzsczewskii*; (**B**) *A. turkestanicum*; (**C**) *A. atrosanguineum*; (**D**) *A. margaritae*; (**E**,**F**) *A. suworowii*. P.s.—*Pisum sativum* internal standard, V.f.—*Vicia faba* internal standard.

According to the data (Table 1), most species demonstrate diploidy (2n = 16), which indicates the predominance of x = 8. For *A. leptomorphum*, *A. parvulum*, and *A. semenowii*, there is no information available on the number of chromosomes. Data on the genome size of these samples were obtained (Table 2 and Figure 3).

### 2.3. Phylogenetic Analysis

**Figure 4:**
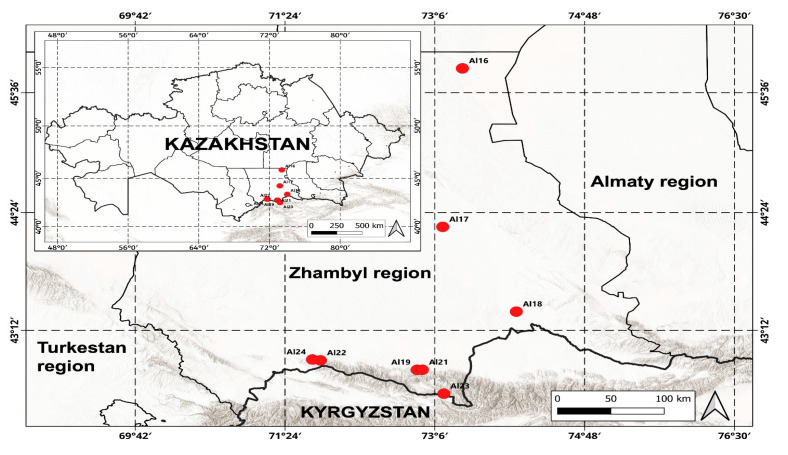
Map of specimen collection points of *Allium* species.

Due to the fact that out of 25 species noted for the Kazakhstan part of the flora of the Kyrgyz Alatau range, we had actual genetic material at our disposal for only 6 species (Figure 4), we were able to obtain ITS sequences only for these 6 species (eight specimens) (Table 1); the remaining ITS sequences were taken from the NCBI database [53]. It should be noted that most of the sequences used in this paper (for the missing species from the NCBI database) were sequenced earlier also by the authors of this paper.

The ITS tree of sequences is made using the studied specimens and supplemented with specimens from the NCBI database [53]. Samples of our own materials are highlighted in bold in the tree. For ease of visual perception, colour highlighting is provided.

**Figure 5:**
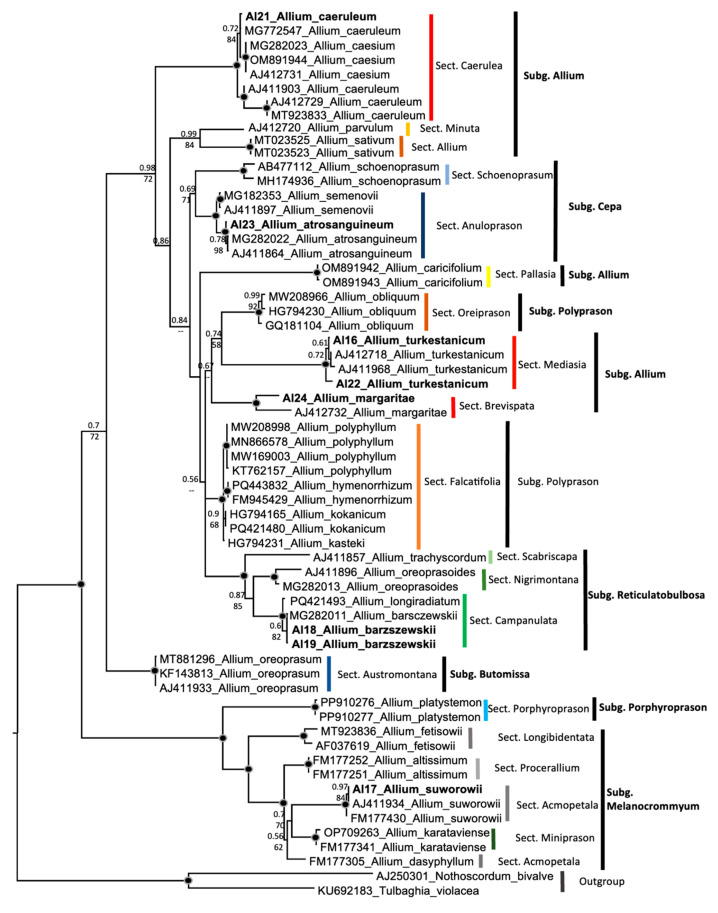
ITS tree of species of the genus *Allium*. The joint presence of Bayesian with a probability greater than 0.98 and bootstrap support greater than 95% is indicated by a black dot. The samples we investigated are highlighted in bold. The following data were obtained by running the data through the JModeltest software: 2.1.10. 010234+G+F, -lnL 7923.16582, AIC 16137.894239, weight 0.493067.

The tree shows not only the species affiliation, but also the subgenus and sectional divisions (Figure 5). For *Allium caeruleum*, two different sequence types are present. Our accession A121 from the Kyrgyz Alatau, along with accession MG772547, is a sister group to *Allium caesium*. However, there are also three accessions in the NCBI GenBank [53], AJ412729, AJ411903, and MT923833, that act as sister groups to *A. caesium* and *A. caeruleum*, respectively (Figure 5). All other accessions of *Allium* taxa from the Kyrgyz Alatau are in the expected position, and each species forms a clear monophyletic clade.

## 3. Discussion

The analysis of species composition shows that within the Kyrgyz Alatau, representatives of subgenera *Allium* and *Melanocrommyum* are the most widely distributed among seven subgenera of the studied genus.

The subgenus *Allium* includes the largest number of species—nine (five sections), belonging mainly to the *Allium* section and characterised by adaptation to growing in the upper belt of mountains (subalpine and alpine belts). Representatives of the subgenus *Allium* are characterised by a wide ecological amplitude and play a key role in the vegetation cover of mountain ecosystems of the Kyrgyz Alatau. The subgenus *Melanocrommyum* is represented by five species (three sections), characterised by characteristic large inflorescences, which underlines their potential ornamental value. Representatives of the subgenus are adapted to arid conditions and are confined to stony places. Phenological plasticity and distinct ecological specialisation indicate a high degree of adaptability of the genus to various altitudinal zones of the region.

The richness of species and their specialisation underline the floristic uniqueness of the Kyrgyz Alatau as an important centre of biodiversity of the genus *Allium* in Central Asia. The majority of species are regionally confined to the Tien Shan floristic province. Some species are also found in the neighbouring ranges: Terskey Alatau, Zailiyskiy Alatau, Karatau [54]. *Allium* species in the Kyrgyz Alatau are clearly differentiated by altitudinal and microclimatic conditions, which indicates their high ecological plasticity and adaptation to extreme mountain conditions. Distribution by belts reflects the gradient from xerophytic steppes to mesophytic high-mountain meadows. Species of the genus *Allium* within the Kyrgyz Alatau in the territory of Kazakhstan demonstrate clear confinement to certain altitudinal belts and habitat types. The majority of species are found in mountain, subalpine, and alpine regions, where they are adapted to particular environmental conditions [13].

According to the results of the analysis by flow cytometry, data were obtained for six species of the genus *Allium*, represented in four subgenera and belonging to different sections. The following species were identified in the subgenus *Allium*: *A. turkestanicum* from section *Mediasia* with DNA content of 22.310 ± 0.566 pg, *A. margaritae* from section *Brevispatha* with 13.907 ± 0.047 pg, and *A. caeruleum* from section *Caerulea* with 20.043 ± 0.796 pg. In subgenus *Melanocrommyum*, *A. suworiwii*, a representative of section Acmopetala, is characterised by a value of 44.159 ± 0.446 pg. The subgenus *Reticulatobulbosa* is represented by the species *A. barzschzewskii* from section *Campanulata* (21.948 ± 0.624 pg), and subgenus *Cepa* by the species *A. artrosanguineum* from section *Annuloprason* with DNA content of 22.740 ± 0.018 pg (Table 2).

In the Plant DNA database [55], for most species characteristic of the Kyrgyz Alatau, the DNA content varies within 20–30 pg, with some exceptions showing much higher values. For example, *Allium karataviense* recorded 39.68 pg, *A. platystemon* 38.80 pg, and *A. suworowii* 37.62 pg. According to Jones and Rees [56], the minimum amount of DNA within the genus *Allium* was previously recorded in *A. schoenoprasum* (16.90 pg). It should be noted that for a significant number of species of the genus *Allium*, the size of the nuclear genome has not been determined so far, which opens prospects for further studies.

Within the framework of the present work, for *A. margaritae*, we recorded the minimum value of DNA content in the genus *Allium* equal to 13.907 ± 0.047 pg (Table 2; Figure 3). This is significantly lower than the value reported in Vakhtina et al. [43], where 31.50 pg is stated for the same species. This discrepancy probably indicates the possible presence of polyploid forms within *A. margaritae*. Considering the low coefficient of variation of our measurements (0.34%), the data obtained seem reliable.

Similar discrepancies between published data and our results were found for *Allium barzschzewskii* and *A. suworowii*. For *A. barzschzewskii*, we determined the DNA content at 21.948 pg, while a number of earlier publications for this species state a value of 31.60 pg [43]. Taking into account outdated methods of analysis and possible methodological differences, a discrepancy of about 10 pg can be considered acceptable.

For *A. suworowii*, we obtained a value of 44.159 pg, whereas the database states 37.62 pg [57,58]. Despite some difference, both values are in a close range and probably reflect both intraspecific variability and differences in the methods used. It should be emphasised that modern flow cytometry technologies provide higher accuracy and reproducibility of results, which increases the reliability of our measurements.

The species *A. caeruleum* showed a genome size of 20.043 pg, which is comparable to the previously published value of 23.50 pg [42]. Notably, the presence of polyploid forms (2n = 24, 32) (Table 1) in *A. caeruleum* indicates the species capacity for polyploidisation, which may be associated with adaptive responses or geographical variability.

For the two species studied, *A. artrosanguineum* and *A. turkestanicum*, the results of flow cytometry are presented for the first time. The values obtained are 22.740 ± 0.018 pg and 22.310 ± 0.566 pg, respectively.

Although the species are most likely diploid, differences in chromosome numbers within the section [59] suggest possible cytogenetic variation.

The discrepancy between our data and that of the published literature may be attributable to instrumental error, variations in the applied standards, and the utilisation of microdensitometry in certain studies.

Molecular phylogenetic data are of particular interest: based on the analysis of ITS-sequences, it was found that the studied onion species from the Kyrgyz Alatau occupy the expected phylogenetic positions and correspond to previously published phylogenetic reconstructions [60,61,62,63,64,65], with barely significant deviations detected. It is interesting that in *Allium caeruleum*, two different sequence types are present. Our accession A121, along with accessions MG772547 and AJ411903, is a sister group to *Allium caesium*. However, there are also two accessions, AJ412729 and MT923833, that act as sister groups to *A. caesium* and *A. caeruleum*, respectively. A broader study of *A. caeruleum* is warranted. *Allium caeruleum* is known to exhibit a ploidy range (see Table 1).

However, given that only 5 of the 25 *Allium* species distributed in the Kyrgyz Alatau were sampled directly from the Kyrgyz range (see Table 2), further sequencing may reveal new or refined phylogenetic relationships.

## 4. Material and Methods

### 4.1. Distribution Analyses

The objects of the present study were species of the genus *Allium* L. of the western part of the Kyrgyz Alatau.

Field studies were conducted during 2024–2025 in the territory of the western part of the Kyrgyz Alatau. The research included route surveys covering various types of habitats. In the course of field work, a targeted collection of herbarium material and samples of representatives of the genus *Allium* L. growing in this territory was carried out [5,6]. Along with this, they were photographed (Figure 2) and geographically referenced using a GPS device (Figure 4).

The identification of the collected materials was based on fundamental works on the genus *Allium* [3,5,6,66], and all recent publications concerning *Allium* species found in the Kyrgyz Alatau were also taken into account [60,61,62,63,64,65].

An areological analysis of *Allium* species was conducted to determine their geographical distribution and areal status within the study area. The geographical affiliation and nature of the range of each species were assessed based on a combination of floristic and areological sources, including fundamental reference books on the flora of Central Asia and neighbouring regions [67,68,69].

Each species was assigned an areological status according to its distribution outside the study area. Endemism was assessed based on information from modern POWO databases.

The herbarium collections of the Institute of Botany and Phytointroduction (AA, Almaty, Kazakhstan) and the Moscow State University named after M.V. Lomonosov (MW, Moscow, Russia) were studied. The names of taxa are provided according to the databases International Plant Names Index (IPNI) [70] and Plants of the World Online (POWO) [2]. Materials from the Plantarium website [14] were also used.

The QGIS 3.34.13 program was used for data mapping (https://qgis.org, accessed on 5 June 2025).

### 4.2. Flow Cytometry

The DNA content was determined by flow cytometry techniques with propidium iodide (PI) staining. Leaves dried with silica gel were used as samples. Samples were chopped as is standard, using a sharp razor blade in LB01 buffer containing PI (50 µg/mL), RNase (50 µg/mL) [71] supplemented with 12 mM sodium thiosulfate, and 1% polyvinylpyrrolidone [72]. The nuclear suspension was filtered through a nylon filter with a pore size of 30 μm. Analyses were performed on a Cytoflex (Beckman Coulter, Inc.) cytometer. Peaks with at least 1000 nuclei and a CV of less than 5% were used for analysis. Histograms were visualised and processed using CytExpert version 2.4 (Beckman Coulter, Inc., Brea, CA, USA). Descriptive statistics were calculated using XLStat (Addinsoft). As an internal standard, we used the *Pisum sativum* ‘Ctirad’, 2C = 9.09 pg and *Vicia faba* ‘Inovec’, 2C = 26.9 pg [71,73].

### 4.3. Phylogenetic Analyses

ITS DNA fragments were sequenced for the species studied of the genus *Allium*. The primers ITS1 (5′-TCCGTAGGTGAACCTGCGG-3′) and ITS4 (5′-TCCTCCTCCGCTTATTGATATATGC-3′) were used for ITS fragments. Polymerase chain reaction was performed in 50 µL of reaction mixture, using the Biomaster HS-Taq PCR-Color 2x PCR kit (Biolabmix LLC, Novosibirsk, Russia) in the following composition—per sample: 25 µL of ready PCR mixture, 21 µL of H_2_O, 1 µL of 10 mM respective primers, 2 µL of total DNA. The amplification protocol was as follows: 95 °C (3 min); 35 cycles: 95 °C (20 s), 57 °C (30 s), 72 °C (30 s); 72 °C (5 min). Amplification products were purified using microcolumns. Sequencing was performed by the Sanger method, using an ABI PRISM 3500 XL sequencer. Sequence chromatograms were manually edited using Chromas Lite 2.1, aligned with ClustalX [74], and manually refined in MEGA 11 software 11.0. 13 [75].

NrITS datasets were analysed separately through Fitch parsimony with the heuristic search option in PAUP version 4.0 b10 [76], with MULTREES, TBR branch swapping, and 100 replicates of random addition sequence. Gaps were treated as missing data. The consistency index (CI) was calculated to estimate the amount of homoplasy in the character set [77]. The most parsimonious trees returned by the analysis were summarised in one consensus tree using the strict consensus method. Bootstrap support (BS) was performed using 1000 pseudoreplicates to assess the support of the clades [78]. Bayesian phylogenetic analyses were also performed using MrBayes 3.1.23 [79]. The sequence evolution model was chosen by following the Akaike information criterion (AIC) obtained from jModelTest2 [80]. Two independent analyses with four Markov chains were run for 10 million generations, sampling trees every 100 generations. The first 25% of the trees were discarded as burn-in. The remaining 150,000 trees were combined into a single dataset, and a majority-rule consensus tree was obtained, along with posterior probabilities (PPs).

Two species were selected as an outgroup: *Tulbaghia violacea* Harv. and *Nothoscordum bivalve* (L.) Britton [65].

## 5. Conclusions

Thus, as a result of field studies, molecular–genetic analysis, flow cytometry, and a critical review of the literature, it has been established that the flora of the genus *Allium* within the Kyrgyz Alatau includes 25 species belonging to seven subgenera and 17 sections. These data represent the most complete taxonomic assessment of *Allium* species diversity in this region to date. As part of the work, the nomenclature of a number of taxa was updated in accordance with modern systematic approaches (for example, *Allium polyphyllum* is treated as a synonym of *A. carolinianum*, and *A. caricifolium* as a synonym of *A. pallasii*), which is important for the harmonisation of regional and global botanical databases.

Analysis of areal data indicates a predominance of mountain species, highlighting the importance of the Kyrgyz Alatau as one of the key centres for the formation and preservation of high-altitude flora in Central Asia. The results of flow cytometry and karyological (the literature data) analysis showed that, with the same, presumably diploid, level of ploidy, representatives of the genus demonstrate wide variability in nuclear genome size.

Phylogenetic analysis confirmed that most of the studied species corresponded to the expected taxonomic relationships, but also revealed potential taxonomic inconsistencies. This highlights the need for further research on representatives of the genus *Allium* in the western part of the Kyrgyz Alatau, which could lead to a refinement of the species composition, the discovery of new species or species not previously recorded in this region, and the exclusion of erroneously identified taxa.

## Figures and Tables

**Table 1:** Karyological data of species of the genus *Allium* growing in the Kyrgyz Alatau.

No.	Species of the Genus *Allium*	Number of Chromosomes	Literature
1	*A. atrosanguineum*	2n = 16	[35]
2	*A. barsczewskii*	2n = 16	[36]
3	*A. caeruleum*	2n = 16, 24, 32 2n = 16; 24 2n = 16	[37,38] [39,40] [41,42]
4	*A. caesium*	2n = 16	[43,44]
5	*A. caricifolium*	2n = 16	[16,43]
6	*A. dasyphyllum*	2n = 16	[40]
7	*A. fetisowii*	2n = 16	[35,40]
8	*A. hymenorhizum*	2n = 16	[35,39,42,43,45]
9	*A. karataviense*	2n = 18	[35,39,40]
10	*A. leptomorphum*	-	-
11	*A. longiradiatum*	2n = 16	[35]
12	*A. margaritae*	2n = 16	[46]
13	*A. obliquum*	2n = 16	[45,47,48]
14	*A. oreoprasoides*	2n = 16	[46]
15	*A. oreoprasum*	2n = 16	[43,49]
16	*A. parvulum*	-	-
17	*A. platyspathum*	2n = 16	[43,50]
18	*A. platystemon*	2n = 16	[17]
19	*A. polyphyllum*	2n = 16	[35]
20	*A. sativum*	2n = 16	[31,43]
21	*A. semenowii*	-	-
22	*A. suworowii*	2n = 16	[43,49]
23	*A. taschkenticum*	2n = 16	[35,51]
24	*A. trachyscordum*	2n = 16	[52]
25	*A. turkestanicum*	2n = 16	[46]

**Table 2:** Collection points of genetic material of species of the genus *Allium*, accessions number of ITS sequences, and DNA content of the studied species.

No.	Sample Number	Species	Location of Material Collection	Voucher Number	ITS	Mean 2C ± SD, pg	CV	N	Std.
1	Al16	*Allium turkestanicum*	Betpakdala floristic region, Zhambyl region, southern part of Betpakdala N 45.839444 E 73.413889	AA0003680	PV915722	22.310 ± 0.566	2.54%	5	P.s.
	Al22	*A. turkestanicum*	Kyrgyz Alatau, Zhambyl region N 42.889830 E 71.801594	AA0003685	PV915719				
2	Al17	*A. suworowii*	Moyinkum floristic area, Zhambyl region, Chui district, close to the road, Moyinkum-Chu highway, sands. Saxaulnik. N 44.257222 E 73.190556	AA0003681	PV915724	44.159 ± 0.446	1.01%	4	P.s., V.f.
3	Al18	*A. barsczewskii*	Moyinkum floristic area, Zhambyl region, Chui district, close to the road on the left side of the road in the town of Chu. N 43.3925 E 74.026111	AA0003682 (Appendix A)	PV915721	21.948 ± 0.624	2.84%	4	P.s.
4	Al19	*A. barsczewskii*	Kyrgyz Alatau, Zhambyl region, T. Ryskulov district, Karakystak village. N 42.792222 E 72.9	AA0003683	PV915720				
5	Al21	*A. caeruleum*	Kyrgyz Alatau, Zhambyl region, T. Ryskulov district, Karakystak. N 42.792222 E 72.9	AA0003684	PV915723	20.043 ± 0.796	3.97%	3	P.s., V.f.
7	Al23	*A. atrosanguineum*	Kyrgyz Alatau, Zhambyl Oblast, Kaskasu gorge. N 42.543611 E 73.203333	AA0003686	PV915718	22.740 ± 0.018	0.08%	3	P.s.
8	Al24	*A. margaritae*	Kyrgyz Alatau, Zhambyl region, Zhambyl district, Ulken Almalysay, southern slope. N 42.898889 E 71.711944	AA0003687 (Appendix A)	PV915717	13.907 ± 0.047	0.34%	3	P.s.

Note: pg—picogram, CV—coefficient of variation sample, N—number of analysed individuals, Std.—standard deviation.

## Data Availability

All data supporting this study’s findings are available in the main text or Appendix A.
